# Network approaches for omics studies of neurodegenerative diseases

**DOI:** 10.3389/fgene.2022.984338

**Published:** 2022-09-16

**Authors:** Na Zhao, Zachary Quicksall, Yan W. Asmann, Yingxue Ren

**Affiliations:** ^1^ Department of Neuroscience, Mayo Clinic, Jacksonville, FL, United States; ^2^ Department of Quantitative Health Sciences, Mayo Clinic, Jacksonville, FL, United States

**Keywords:** network, omics, multi-omics integration, Alzheimer’s disease, neurodegenerative disease

## Abstract

The recent methodological advances in multi-omics approaches, including genomic, transcriptomic, metabolomic, lipidomic, and proteomic, have revolutionized the research field by generating “big data” which greatly enhanced our understanding of the molecular complexity of the brain and disease states. Network approaches have been routinely applied to single-omics data to provide critical insight into disease biology. Furthermore, multi-omics integration has emerged as both a vital need and a new direction to connect the different layers of information underlying disease mechanisms. In this review article, we summarize popular network analytic approaches for single-omics data and multi-omics integration and discuss how these approaches have been utilized in studying neurodegenerative diseases.

## Introduction

Human diseases have enormous levels of complexity. The vast majority of conditions, such as Alzheimer’s disease (AD), are caused by a combination of genetic, environmental, and lifestyle factors, most of which have not yet been identified (Knopman et al., 2021). To understand this disease complexity and explore the undefined risk factors, the research field has tended to employ the single- or multi-omics approaches that aim to study the broader biological processes in an unbiased way, instead of focusing on single molecules. This rapid progress in basic science brought by omics study seems to be quite fruitful; however, how to extract meaningful insights from these large-scale and high-dimensional data sets from multiple sources is still a challenge.

Network-based approaches to studying human disease have had promising applications. Networks constitute the foundation of biological systems. A network can involve different levels of biological entities that connect to one another through direct or indirect interactions. The central idea of applying network analysis is to reduce the dimensionality of data from thousands of altered genes, proteins, metabolites, lipids, or other biological entities to a smaller and more interpretable set of altered processes (Pe’er and Hacohen, 2011).

The fast development of omics technology combined with a decrease in cost has made multi-omics readily available to researchers. These different omics data types are unique but complementary with each set containing information that is not represented in others. Taken together as a collective group, these various omics representations constitute the biological networks that drive disease mechanisms. Multi-omics integration using a network approach helps to bridge the gap between genotype and disease, which remains an essential task in the current era of big data analytics. In the following sections, we summarize popular network analytic approaches for omics studies of neurodegenerative diseases from the standpoints of both single-omics and multi-omics integration.

## Common network approaches for single-omics

**FIGURE 1:**
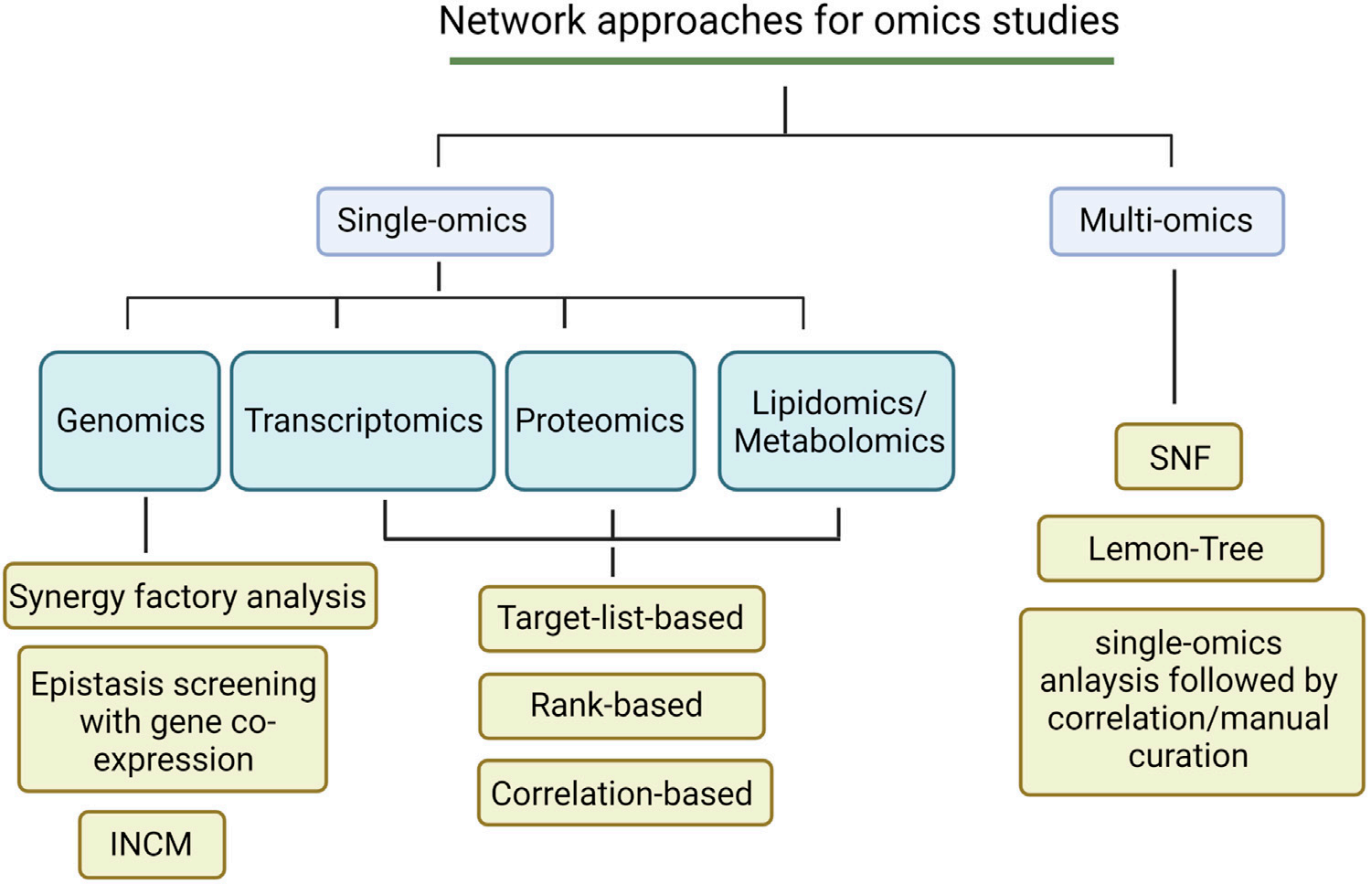
An overview of network analytic approaches for omics studies.

As most neurodegenerative disease studies focused on the analysis of single-omics for many years, successful network approaches have been developed to fit the data type. In fact, network frameworks can often be adapted to fit multiple data types. Here, we summarize several common network approaches for single-omics analysis [Figure 1] (Created with BioRender.com).

### Genomics

Genomics analysis utilizing whole exome or whole genome sequencing is perhaps the most common omics for genetic studies aiming to identify risk and protective mutations (Pottier et al., 2019). However, despite the success of genome-wide association studies (GWAS) for human disease, a large portion of genetic variance remains unexplained (Manolio et al., 2009; Zuk et al., 2012). Gene-gene interactions, such as epistasis, may explain a portion of the missing variance. For example, Combarros et al. (2009) used synergy factor analysis to assess epistasis in sporadic AD, providing a measure of both the size and significance of interactions between genetic variants. They identified significant gene-gene interactions associated with AD that were involved in networks of cholesterol metabolism, β-amyloid (Aβ) metabolism, inflammation, oxidative stress, and others. More recently, Reddy et al. (2020) analyzed close to ten thousand whole exomes from the Alzheimer’s Disease Sequencing Project and used polygenic risk scores to combine single nucleotide variant analysis with co-expression network analysis to identify four genes significantly associated with AD within a co-expression network with *TREM2*. Another recent study combined epistasis screening with co-expression network analysis using multiple independent datasets and identified two significant genetic interactions implicated in AD (Wang et al., 2021).

Notably, the Individualized Network-based Co-Mutation (INCM) methodology was developed for quantifying the putative genetic interactions in cancer. This approach has been demonstrated to uncover differential genetic interaction burdens between mutated or known cancer genes compared to other essential genes. The network-predicted putative genetic interactions were also correlated with patient survival (Liu et al., 2020). While this approach has only been applied to cancer studies thus far, it could be adapted to other disease studies for the comprehensive identification of candidate therapeutic pathways.

### Transcriptomics

Transcriptomics has evolved from the traditional bulk RNA sequencing to single cell/single nuclei RNA sequencing (sc/snRNA-seq) allowing the molecular mechanism of the disease to be directly measured and explored at the cellular level. More recent spatial transcriptomics technologies measure cell-level expression activity in their morphological context. In the context of AD, it has been demonstrated that the strongest disease-associated transcriptomic changes appeared early in pathological progression and were highly cell-type specific, whereas genes upregulated at late stages were common across cell types and primarily involved in the global stress response (Mathys et al., 2019). Regardless of the underlying technological differences of these modalities, the resulting transcriptomics data lend themselves to the application of target-list-based, rank-based, and correlation-based network analysis.

Target-list-based approaches rely on a list of specific gene targets, often selected from differentially expressed genes between conditions, to identify enriched gene ontologies, canonical pathways, and regulatory networks. This approach is intuitive, and the gene targets identified can often be validated in wet labs (Ren et al., 2018; Zhao et al., 2020a; Zhao et al., 2020b). However, when gene expression differences between conditions are subtle, the target-list approach will not work well because very few genes will pass reasonable statistical significance thresholds for inclusion in downstream analyses. Alternatively, a rank-based approach, such as Gene Set Enrichment Analysis (GSEA), can circumvent this limitation and find the biological significance behind small but concerted changes. Instead of evaluating the contribution of individual genes, GSEA focuses on gene sets, which come in the form of published pathways, ontologies, or a specific curation to assess whether members of a gene set tend to occur toward the top or bottom of the ranked gene list, in which case the gene set is regarded as being correlated with the trait of interest (Subramanian et al., 2005). A recent AD study applying GSEA to snRNA-seq data of a transgenic mouse model of 5x familial Alzheimer’s disease (5xFAD) found that a specific subtype of AD-associated excitatory neuron possessed downregulated AD pathways at an early stage of disease progression (Shao et al., 2021), a finding which highlights the value of applying network approaches traditionally used on bulk data to the more sparse representations found at the single-nuclei level.

Correlation-based analysis, such as Weighted Gene Co-expression Network Analysis (WGCNA), relies on calculating correlation coefficients between gene pairs, grouping highly correlated genes into modules (i.e., subnetworks), and then correlating the expression pattern of the module with traits of interest. The module hub genes, those with the highest connectivity to all genes within the module, can be nominated as biomarkers or therapeutic targets. For example, Liang et al. (2018) investigated co-expression networks in AD and control samples of the human hippocampus and found that mineral absorption, NF-κB signaling, and cGMP-PKG signaling pathways were associated with AD clinical severity. Analysis of human temporal cortex and cerebellum samples by our group found that TMEM106B protective haplotypes were associated with gene networks involved in synaptic transmission. Separately, the risk haplotype was associated with gene networks enriched for immune response (Ren et al., 2018). Our group also studied the brain transcriptomics of human apolipoprotein E (apoE)-targeted replacement mice across different ages and sex and reported an aging-related immune module led by *Trem2* and *Tyrobp*, and an apoE-related module with multiple *Serpina3* genes (Zhao et al., 2020b). Notably, the different network approaches mentioned here are complementary, and a study can often benefit from applying more than one approach to gain different insights (Rexach et al., 2020) (Ren et al., 2018; Zhao et al., 2020a; Zhao et al., 2020b).

Finally, it is worth noting that while hub genes are often key regulators in gene networks identified by methods such as WGCNA, the definition of hub genes is not confined to WGCNA. Indeed, the presence of hubs seems to be a general feature of all cellular networks. For example, transcription factors (TF) are often regarded as hub genes because they bind to specific DNA sequences, form interactions with many genes, and conduct most of the regulation activities in the human genome, Additionally, cancer genes such as oncogenes and tumor suppressors are often hub genes in the tumor genetics network (Yu et al., 2017). While hub genes in biological studies are often identified based on highest connectivity (Langfelder and Horvath, 2008), other methods can also be used to name hub genes, such as Kleinberg (1998) score, superior node degree and other node centrality measures (Freeman, 1977; Freeman et al., 1979; Strogatz, 2001; Li et al., 2018; Ruffini et al., 2022).

### Proteomics

Proteomics analyses can utilize many of the same network approaches as transcriptomics due to the easy mapping of genes to proteins (Zhang et al., 2018). Of note, Protein-Protein Interaction (PPI) is one of the main categories of biological networks which form the backbone of signaling pathways, metabolic pathways, and cellular processes required for the normal functioning of cells (Westermarck et al., 2013). The mapping of protein interactions over time has been curated and stored in several large databases, which currently serve as the knowledge base for network analysis of proteomics. Some of the most commonly used databases include Human Protein Reference Database (HPRD) (Peri et al., 2004), Molecular Interaction Database (MINT) (Chatr-aryamontri et al., 2007), Biological General Repository for Interaction Database (BioGRID) (Stark et al., 2006), and the IntAct molecular interaction database (IntAct) (Hermjakob et al., 2004).

Many major studies have relied on protein networks to investigate disease biology. In one multicenter consortium study, the investigator analyzed more than 2,000 human brain tissues via quantitative mass spectrometry (MS)-based proteomics and found a protein network module linked to sugar metabolism emerged as one of the protein modules most significantly associated with AD pathology and cognitive impairment. This module was enriched in AD genetic risk factors as well as microglia and astrocyte protein markers associated with an anti-inflammatory state, suggesting that the biological functions it represents serve a protective role in AD (Johnson et al., 2020). Further proteome and transcriptome comparison revealed that the levels of RNAs and proteins were only partially correlated. This supports the belief that the transcriptome is often not an accurate indicator of protein abundance, because protein abundance can be regulated by posttranscriptional events such as protein turnover. For example, in another study, the investigators analyzed the proteomes of more than 1,000 brain tissues across various cohorts and brain regions to reveal new AD-related proteins. They found that nearly half of the protein co-expression modules, including modules significantly altered in AD such as the MAPK signaling and matrisome-related modules, were not observed in RNA networks from the same cohorts and brain region (Johnson et al., 2022). These results suggest that future studies should consider the integration of transcriptomics with proteomics profiles to better understand the disease.

### Lipidomics and metabolomics

The determination of interactions between individual lipid species or metabolites with other lipids, proteins, and metabolites in the system adds crucial information on disease regulation. The network approach for lipidomics and metabolomics can also be adapted to those commonly used in transcriptomics or proteomics. For example, using a correlation-based network approach, Aikawa et al. (2021) reported that LPS administration and *ABCA7* haplodeficiency affected glycerophospholipid metabolism, linoleic acid metabolism, and α-linolenic acid metabolism. Our group found that aging regulated mouse serum metabolomics and affected networks involving long chain fatty acids, amino acids, and biogenic amines (Zhao et al., 2020b). Köberlin et al. (2015) reported coregulated lipids associated with different immune stimulations, which were conserved in cell lines, mice, and humans. In the same study, gene expressions were mapped to known sphingolipid metabolic pathways to show immune regulations of both transcriptomics and lipidomics. This study, among many others, showcased how lipidomics can be analyzed similarly in terms of the network approach and that connecting different omics through networks is not only helpful but also necessary to understand the disease biology better.

## Network-based multi-omics integration methods

Current multi-omics integration methods can be classified into two general categories: statistical integration and network-based integration. Statistical integration relies on drawing inferences from the data themselves without using prior knowledge. For example, a top-performing statistical integration method is joint dimensionality reduction (jDR), and many tools using this concept have been reviewed (Cantini et al., 2022). Network-based integration, on the other hand, views biological systems as interconnected entities, with each omics contributing to revealing the true connections of the networks. The central idea is built on the known relationship among these entities, which provides a guide to the type of integration.

The Similarity Network Fusion (SNF) method is one of the few computational network approaches for multi-omics integration. It constructs a sample-similarity network for each data type and integrates these networks into a single comprehensive similarity network via a nonlinear combination method. During this process, weak similarities are removed while strong similarities are added, making the combined network more coherent and robust. SNF has been shown to work well to integrate mRNA, miRNA, and DNA methylation data for five cancer datasets (Wang et al., 2014). Lemon-Tree, a method based on module network inference, was developed to reconstruct co-expression modules and their upstream regulatory programs from multi-omics datasets. On one hand, it finds co-expressed clusters from the gene expression data; on the other hand, it combines other omics data types, such as miRNA expression, copy number variants (CNV), and methylation with the gene module to infer a regulatory score. This approach has shown accurate prediction results from integrating somatic CNV and gene expression levels measured in glioblastoma samples from The Cancer Genome Atlas (TCGA) (Bonnet et al., 2015). However, both approaches are yet to be utilized in neurodegenerative disease studies.

While the true computational network approaches for multi-omics integration are under-developed, many studies that successfully drew valuable conclusions from multi-omics data used network analysis differently. For example, Nativio et al. (2020) used a step-wise approach to integrate transcriptomic, proteomic, and epigenomic data of postmortem human brains in AD and control samples. They first performed transcriptomics analysis and identified differentially expressed chromatin-regulated genes in AD. From there, they investigated proteomics and focused on histone post-translational modifications (PTMs), which are known chromatin regulators. The PTMs of interest were then examined using chromatin immunoprecipitation sequencing (ChIP–seq). Genes associated with these PTM changes were used for pathway enrichment analysis, leading to the conclusion that H3K27ac and H3K9ac were potential epigenetic drivers of AD, which spur disease pathways through dysregulation of transcription and chromatin–gene feedback loops. A more recent study analyzed cerebrospinal fluid (CSF) from individuals with normal cognition or with cognitive impairment. They first analyzed proteomics, metabolomics, lipidomics, and one-carbon metabolism separately, and then integrated them using multi-omics factor analysis (MOFA) (Argelaguet et al., 2018), followed by pathway enrichment analysis. They identified interactions between single-omics modalities and revealed overrepresentation of the hemostasis, immune response, and extracellular matrix signaling pathways in association with AD (Clark et al., 2021). Another group integrated miRNA, total RNA, and proteomics generated from human post-mortem midbrains, and found enriched pathways associated with Parkinson’s disease (PD), including neuroinflammation, mitochondrial dysfunction, and defects in synaptic function. Like the other studies, their approach relies on first analyzing each single-omics; the integration of multi-omics occurs afterward by exploring interaction databases (Caldi Gomes et al., 2022).

Neurodegenerative diseases share many common features such as the accumulation of misfolded proteins and the progressive loss of neurons. These events are usually regulated on multiple omics’ levels (Ruffini et al., 2022). To compare different neurodegenerative diseases, a recent meta-study integrated genomics, transcriptomics, proteomics, and methylomics across four different neurodegenerative disorders, and used network approaches to uncover biological processes both common and unique to the diseases investigated. They found that the four neurodegenerative diseases did not differ in terms of the disease hallmarks, including cell cycle, autophagy and apoptosis, extracellular matrix organization, development, signal transduction/transport, immune system, and metabolic processes. The integration of multi-omics data was done through the intersection of genes from different omics, multi-omics conformity, and protein-protein interaction networks. Some hub genes that were identified through the protein-protein interaction networks are common in different diseases, such as *HDAC1, BIN1, PICALM*, and *APOE.* Interestingly, the involvement of *HDAC1,* which contributes to epigenetic silencing of active chromatin, was found to be the hub gene in transcriptomics data of all four diseases, suggesting a crucial role of epigenomics in unveiling the mechanisms of neurodegenerative diseases (Ruffini et al., 2022).

## Conclusion

In this review, we summarized several common network analysis approaches used in the studies of neurodegenerative diseases. The network approaches for different omics are mainly built on the assumption that the biological entities that function in the same network or pathway correlate. As a result of this assumption, network methods initially designed for a particular type of omics can be adapted to other omics modalities, often with minimal difficulty. At the same time, multi-omics integration approaches have been utilized in cancer studies for over a decade, thanks to the large magnitude of data types available in cancer databases (The Cancer Genome Atlas Network, 2012). Multi-omics integration for neurodegenerative diseases is in its infancy and just beginning to benefit from the availability of larger volumes of multi-omics data. While many studies have gained critical insight from multi-omics data, accurate computational network analytic approaches for multi-omics integration are still developing. It will require continuing refinement as the field evolves, hoping to shed light on our understanding of the complex disease molecular mechanisms and guide future research to find the targeted therapeutic strategies for these devastating neurodegenerative diseases.
